# The effect of diabetes on surgical versus percutaneous left main revascularization outcomes: a systematic review and meta-analysis

**DOI:** 10.1186/s13019-022-01795-w

**Published:** 2022-04-01

**Authors:** Marc-André d’Entremont, Ryuichiro Yagi, Soziema J. S. Salia, Shuqi Zhang, Lamyaa Shaban, Yakubu Bene-Alhasan, Stefania Papatheodorou, Étienne L. Couture, Thao Huynh, Michel Nguyen, Rikuta Hamaya

**Affiliations:** 1 Division of Cardiology, Department of Medicine, Sherbrooke University Hospital Center (CHUS), 3001, 12e Avenue Nord, Sherbrooke, QC J1H 5N4 Canada; 2 Harvard T.H. Chan School of Public Health, Boston, MA USA; 3 Saiseikai Central Hospital, Tokyo, Japan; 4 McGill Health University Center, Montreal, QC Canada; 5 Brigham and Women’s Hospital, Boston, MA USA

**Keywords:** Diabetes, Drug-eluting stents, Coronary artery bypass graft, Left main coronary artery disease, Percutaneous coronary intervention

## Abstract

**Background:**

The optimal method of coronary revascularization for diabetes mellitus (DM) patients with left main coronary artery disease (LMCAD) is controversial in the drug-eluting stent (DES) era.

**Methods:**

We performed a systematic review and meta-analysis comparing DES-based percutaneous coronary intervention (PCI) to coronary artery bypass grafting (CABG) for LMCAD in DM patients and tested for effect measure modification (EMM) by diabetes for adverse events. We included all randomized controlled trials (RCTs) and observational studies comparing CABG to DES-based PCI including DM patients with LMCAD published up to March 1, 2021. We completed separate random-effects meta-analyses for four RCTs (4356 patients, mean follow-up of 4.9 years) and six observational studies (9360 patients, mean follow-up of 5.2 years).

**Results:**

In RCTs among DM patients, DES-based PCI, compared to CABG, was associated with a 30% increased relative risk (RR) (RR 1.30, 95% CI 1.09–1.56, I^2^ = 0%), while among non-DM patients, there was a 25% increased relative risk (RR 1.25, 95% CI 1.07–1.44, I^2^ = 0%) for the composite endpoint of all-cause mortality, myocardial infarction, stroke, and unplanned revascularization (MACCE). There was no evidence of EMM (*p*-value for interaction = 0.70). The mean weighted SYNTAX score was 25.7. In observational studies, there was no difference between DES-based PCI and CABG for all-cause mortality in patients with DM (RR 1.13, 95% CI 0.91–1.40, I^2^ = 0%).

**Conclusions:**

CABG was superior to PCI for LMCAD in RCTs in DM patients for MACCE. Heart teams may consider DM as one of the many components in the clinical decision-making process, but may not want to consider DM as a primary deciding factor between DES-based PCI and CABG for LMCAD with low to intermediate anatomical complexity in the other coronary arteries.

***Study registration*:**

CRD42021246931 (PROSPERO).

**Supplementary Information:**

The online version contains supplementary material available at 10.1186/s13019-022-01795-w.

## Background

Diabetes mellitus (DM) is a major risk factor for coronary artery disease and is also a predictor of adverse cardiovascular events after both coronary artery bypass grafting (CABG) and percutaneous coronary intervention (PCI) in patients with coronary artery disease [1]. Several trials have demonstrated CABG to be superior to PCI in DM patients with multivessel disease without left main coronary artery disease (LMCAD) [2, 3]. However, evidence regarding the treatment strategy in patients with LMCAD has been sparse, as CABG had traditionally been the treatment of choice in the absence of significant contraindications [4]. With the widespread use of drug-eluting stents (DES) and the progress in the PCI technique, several recent trials have demonstrated DES-based PCI to be equivalent to CABG for LMCAD regarding the composite endpoint of long-term all-cause mortality, cardiovascular death, and myocardial infarction [5–9]. The revascularization guidelines recommend both CABG and PCI for LMCAD with low-to-intermediate anatomical complexity—however, they lack specific recommendations for LMCAD patients with DM [1, 10]. Evidence regarding whether DM status should be accounted for when choosing a revascularization strategy for LMCAD is unclear [11]. A recent meta-analysis included bare-metal stents (BMS), did not include the most relevant clinical trials and did not restrict to only LMCAD [12]. Considering the clinical equipoise for the optimal revascularization strategy comparing DES-based PCI versus CABG for LMCAD in DM patients, the unclear potential effect-measure modification (EMM) by diabetes, and the lack of adequately powered studies, an updated meta-analysis would be timely and significant. Therefore, we leveraged the recent addition of new long-term data from multiple randomized controlled trials (RCT) for LMCAD with DM subgroups to perform a systematic review and meta-analysis [6, 9]. We also included observational studies to provide an overview of the totality of the evidence.

## Methods

We registered the present meta-analysis in the PROSPERO international prospective register of systematic reviews (CRD42021246931) and followed the PRISMA guidelines (Additional file 1: Table S1) [13].

### Search strategy

We completed a systematic search of Pubmed, Embase, and the Cochrane Central Register of Controlled Trials from January 1, 1999, to March 1, 2021. We restricted our search to 1999 as this was the year of the first-in-man drug-eluting stent implantation [14]. Additional file 1: Table S2 denotes our search strategy. We also hand-searched the bibliographies of the most recent and relevant meta-analyses to identify other potentially eligible studies [5, 12, 15]. Two independent authors performed the search (MD and LS), and 6 authors (YB, MD, LS, SS, RY, and SZ) performed the literature review using the Covidence platform (Covidence systematic review software, Veritas Health Innovation, Melbourne, Australia). Two of the 6 authors (YB, MD, LS, SS, RY, or SZ) independently reviewed each study, and disputes were resolved by consensus following discussion with 1 of 2 authors (MD and RY) who was not involved in the conflict.

### Inclusion and exclusion criteria

All published RCTs and observational studies directly comparing DES-based PCI to CABG with effect estimates available for patients with DM and LMCAD were considered eligible. All studies were required to have more than 1 year of follow-up to allow for a meaningful accrual of events. Observational studies were included if they provided adjusted estimates by either propensity score methods or multivariable outcome regression. The study with the longest follow-up was used if multiple studies were published based on the same study population. We excluded all studies that only had effect estimates using BMS.

### Endpoints

The endpoint for the analysis of RCTs was the composite endpoint of all-cause mortality, myocardial infarction, stroke, or unplanned revascularization (MACCE) which was available in all included RCTs. Of note, individual components of MACCE were not available for patients with and without DM. A summary of definitions of the outcomes in the included RCTs is provided (Additional file 1: Table S3). Three endpoints for the observational studies were compiled: (1) all-cause mortality, (2) the composite endpoint of all-cause mortality, myocardial infarction, or stroke, and (3) unplanned revascularization.

### Data extraction and risk of bias

The number of CABG and DES-based PCI patients, the number of patients with and without DM, age, follow-up time, the proportion of patients having an acute coronary syndrome (ACS) as the indication for revascularization, coronary artery disease severity, stent type, and relevant endpoints were extracted once by 1 of 5 authors (BY, LS, SS, RY, and SZ). A sixth author (MD) extracted all data independently a second time. Included RCTs were assessed for bias using Cochrane’s Collaboration risk-of-bias tool [16]. We used the ROBINS-I tool for the observational studies to evaluate study quality [17].

### Statistical analysis

For the randomized trials, the outcome was analyzed on an intention-to-treat basis, and intervention and control groups were used to compute relative risks (RR). For observational studies, the adjusted hazard ratios were approximated to RR under the assumption of rare outcomes [18]. We performed random-effects meta-analyses with the restricted maximum likelihood estimator using the DerSimonian-Laird method separately for the RCTs and observational studies. To assess EMM, we compared the pooled estimates from the DM patients to the non-DM patients and performed subgroup analyses for interaction.

We used the I^2^ statistic to evaluate heterogeneity [19]. The extent of heterogeneity was considered low, moderate, substantial, and considerable if the I^2^ statistic was 0–40%, 30–60%, 50–90%, and 75–100%, respectively. To examine the robustness of our findings, we performed sensitivity analyses: (1) fixed-effect meta-analysis for the RCTs and (2) influence analysis for the observational studies. Furthermore, we examined for funnel plot asymmetry to detect potential publication bias and used Egger’s test to detect small study effects only for the observational studies due to the number of studies.[20, 21] Median and interquartile ranges were transformed into weighted means (weighted by the number of study participants) as appropriate using the method described by Wan et al.[22]

We also performed meta-regression by follow-up time as a continuous variable, by the proportion of three-vessel disease, and by the proportion of patients who had an acute coronary syndrome (ACS) as the indication for revascularization for the composite of all-cause mortality, myocardial infarction, and stroke in the observational studies. Statistical significance was set at a threshold of *p* < 0.05, and 95% confidence intervals (CI) were computed. All analyses were performed using the *metan* package in Stata version 16.1 (StataCorp, College Station, TX, USA).

## Results

**Fig. 1:**
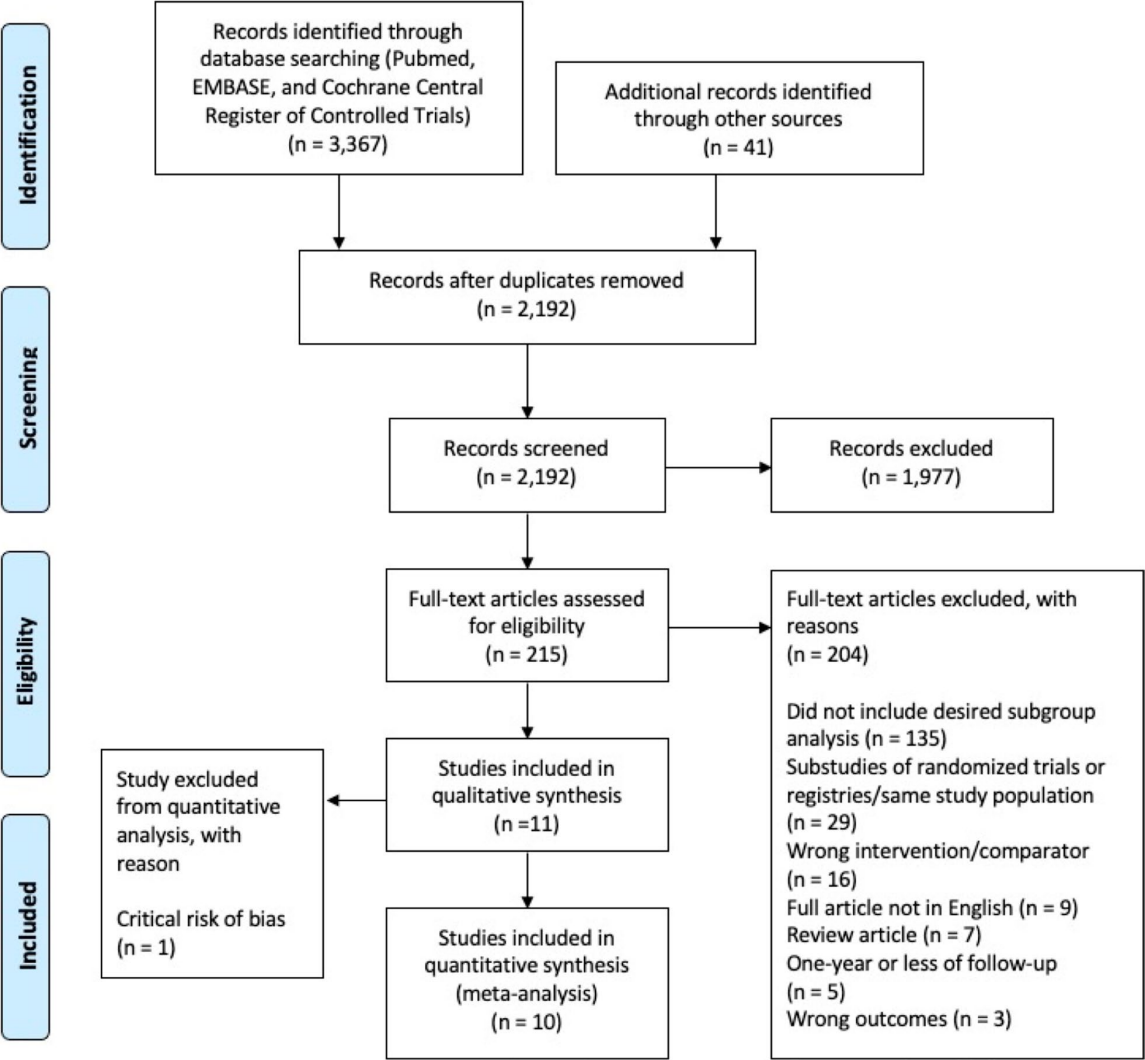
PRISMA flowchart

Figure 1 (PRISMA flowchart) describes the selection of studies for the analysis. The characteristics of the included studies are summarized in Table 1. Four RCTs containing 4356 patients with a mean weighted follow-up of 4.9 years were included [6, 7, 9, 23]. Among the RCTs, 2186 patients (50.2%) were treated by DES-based PCI, and 1080 patients (24.8%) had DM. The mean weighted age was 65.5 years, and the mean weighted SYNTAX score was 25.7. The total number of accrued MACCE events across the four RCTs was 1058, of which 331 (31.3%) were in the DM groups and 727 (68.7%) in the non-DM group.

**Table 1:** Study characteristics

Author	Years	Number of DES/CABG patients	Number of DM/non-DM patients	Age* (years)	Follow-up (years)	ACS (%)	Coronary artery disease severity	Entry criteria	Stent type	Primary outcome	Adjusting method	Risk of bias
*Randomized controlled trials*												
Morice et al.	2014	346/322	150/518	65.5 (9.9)	5 (mean)	29.8	Mean SYNTAX score (core-lab): 29.6 DES/30.2 CABG	≥ 50% ULMCA visual stenosis Silent ischemia or stable/unstable angina	Paclitaxel	Composite of all-cause mortality, stroke, MI, or repeat revascularization at 5-year follow-up	Randomization	Some concern
Milojevic et al.	2019	948/956	554/1350	65.9 (9.6)	3 (mean)	39.2	Mean SYNTAX score (core-lab): 26.9 DES/26 CABG	≥ 70% ULMCA visual stenosis, 50–70% stenosis if significant by invasive or non-invasive testing, SYNTAX ≤ 32 Silent ischemia, angina, or ACS	Everolimus	Composite of all-cause mortality, MI, or stroke at median 3-year follow-up	Randomization	Some concern
Holm et al.	2020	592/592	184/1000	66.4 (9.7)	4.9 (median)	17.4	Mean SYNTAX score (core-lab): 22.5 DES/22.4 CABG	≥ 50% ULMCA visual stenosis or FFR ≤ 0.8 Angina, ACS ≤ 3 additional non-complex lesions	Biolimus	Composite of all-cause mortality, stroke, non-index treatment-related MI or unplanned revascularization at 5 years or until 275 events	Randomization	Some concern
Park et al.	2020	300/300	192/408	62.3 (9.8)	11.3 (median)	50.5	Mean SYNTAX score (core-lab): 22.4 DES/25.8 CABG	≥ 50% ULMCA visual stenosis Silent ischemia, angina, NSTEACS	Sirolimus	Composite of all-cause mortality, MI, stroke, or ischemia-driven revascularization at 10-year follow-up	Randomization	Some concern
*Observational studies*												
Zhao et al.	2011	56/116	172/0	61 (N/A)	2.4 (median)	98.3	LMCAD + 3-vessel disease: 46.4% DES/73.3% CABG	ULMCAD (severity unspecified) Medically treated DM patients	Sirolimus (94.6%), Zotarolimus (5.4%)	All-cause mortality	Multivariable outcome regression	Serious
Meliga et al.	2013	520/306	826/0	66.9 (9.7)	4.0 (mean)	66.8	LMCAD + 3-vessel disease: 38.1% DES/72.9% CABG	ULMCAD (severity unspecified) Medically treated DM patients	Sirolimus (57.9%), Paclitaxel (40.0%), Zotarolimus (0.2%), Everolimus (1.9%)	Composite endpoint of death, myocardial infarction, or stroke	Propensity score with outcome regression	Moderate
Yu et al.	2015	465/457	274/648	62.8 (N/A)	7.1 (median)	81.0	LMCAD + 3-vessel disease: 35.3% DES/67.2% CABG	≥ 50% ULMCA visual stenosis	DES unspecified	Composite endpoint of death, myocardial infarction, or stroke	Multivariable outcome regression	Serious
Zheng et al.	2016	1442/2604	1154/2892	61.4 (98)	3 (mean)	52.3	LMCAD + 3-vessel disease: 34.5% DES/78.7% CABG	≥ 50% ULMCA visual stenosis	DES unspecified	All-cause mortality at 3-years	Propensity score with outcome regression	Moderate
Lee et al.	2017	950/950	736/1164	64.6 (9.8)	4.7 (median)	58.0	LMCAD + 3-vessel disease: 60.9% DES/61.4% CABG	≥ 50% ULMCA visual stenosis	DES unspecified	Composite endpoint of death, myocardial infarction, or stroke	PSM	Moderate
Lee et al.	2020	804/690	507/987	62.1 (10.6)	12 (median)	72.1	LMCAD + 3-vessel disease: 34.0% DES/94.1% CABG	≥ 50% ULMCA visual stenosis	DES unspecified	All-cause mortality	IPTW	Moderate

ACS, Acute coronary syndrome; CABG, Coronary artery bypass surgery; DES, Drug-eluting stents; DM, Diabetes mellitus; IPTW, inverse probability of treatment weighting; NSTEACS, Non-ST elevation acute coronary syndrome; PSM, propensity score matching; ULMCA, Unprotected left main coronary artery; ULMCAD, Unprotected left main coronary artery disease

*Age converted from median into means using the method described by Wan et al. when appropriate [24]

Furthermore, a total of 6 observational studies, including 9360 patients with a mean weighted follow-up of 5.2 years were analyzed [24–29]. Of these total patients, 4237 patients (45.3%) were treated by DES-based PCI, and 3669 (39.2%) had DM. The mean weighted age was 62.8 years. Furthermore, 1745 of 4237 DES patients (41.2%) compared to 3898 of 5123 CABG patients (76.1%) had LMCAD plus 3-vessel disease, respectively. Patients who underwent CABG had a significantly more extensive coronary artery disease than DES-treated patients in all but one study (Lee et al., 2017). No clear trend emerged for age and ACS presentation.

### Risk of bias evaluation

The risk of bias evaluation is summarized in Additional file 1: Tables S4 and S5. We evaluated all the RCTs as having ‘some concern’ for bias. The ‘measurement of outcome’ domain was judged to be of ‘some concern’ for the Morice et al. [7], the Milojevic et al. [23], and the Holm et al. [6] studies as blinding of the adjudication committee was unspecified. The ‘randomization process’ domain was judged to be of some concern for the Park et al. [9] study as this was the only RCT where the randomization was not stratified for LMCAD and diabetes.

We evaluated the observational studies as having a ‘moderate’ to ‘serious’ risk of bias. Two of the smaller studies (Zhao et al. [24] and Yu et al. [26]) were judged to be at serious risk of overall bias because of suboptimal confounding adjustment, and all studies were deemed to be minimally at moderate risk of bias for participant selection because of confounding by indication bias [24, 26]. One study was excluded due to ‘critical’ risk of bias because the adjustment for confounding was judged to be insufficient [30].

### Randomized controlled trials

**Fig. 2:**
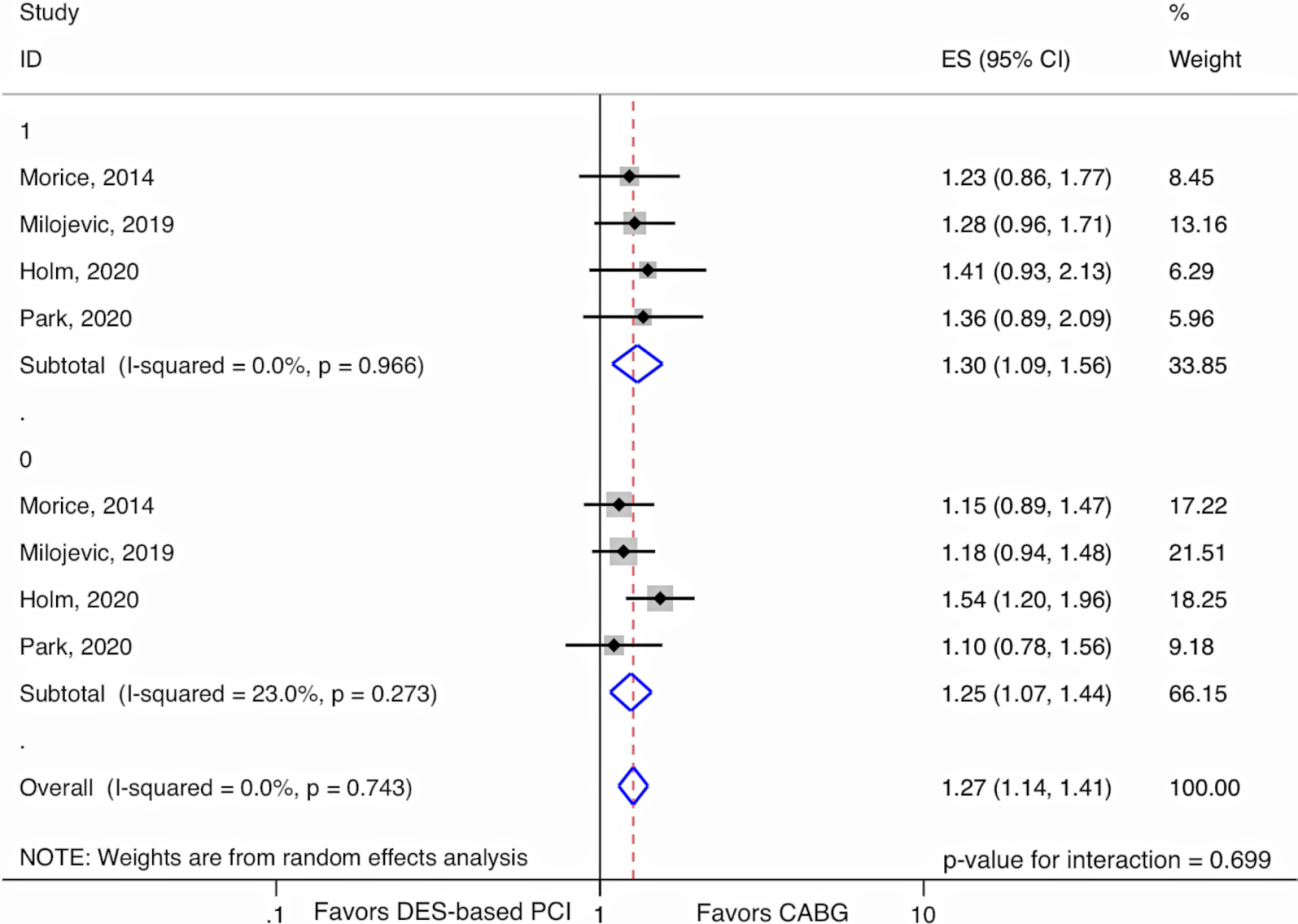
Random-effects meta-analysis testing for effect measure modification by diabetes comparing DES-based PCI to CABG using relative risks for the composite endpoint of all-cause mortality, myocardial infarction, stroke, or unplanned revascularization in RCTs. 1 = DM, 0 = non-DM; CABG, Coronary artery bypass graft; CI, Confidence interval; DES, Drug-eluting stents; ES, Estimate

At the longest follow-up, DM patients who underwent DES-based PCI with DES had a 30% relative increase (RR = 1.30, 95% CI 1.09–1.56, *p*-value = 0.004) in MACCE compared to those who underwent CABG for LMCAD (I^2^ = 0.0%, *p*-value = 0.97) (Fig. 2). Non-DM patients treated by DES-based PCI experienced a 25% relative increase (RR = 1.25, 95% CI 1.07–1.44, *p*-value = 0.004) in MACCE compared to patients treated by CABG (I^2^ = 23.0%, *p*-value = 0.27). There was no evidence of EMM by diabetes (*p*-value for interaction = 0.70).

### Observational studies

**Fig. 3:**
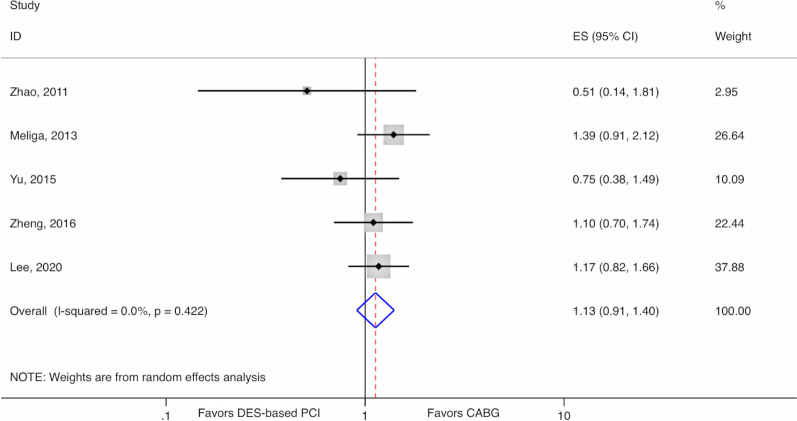
Random-effects meta-analysis for diabetic patients comparing DES to CABG using relative risks for all-cause mortality in observational studies. CABG, Coronary artery bypass graft; CI, Confidence interval; DES, Drug-eluting stents; ES, Estimate

Using the five studies with available individual death outcomes, there was no difference between DES-based PCI and CABG for DM patients regarding all-cause mortality (RR = 1.13, 95% CI 0.91–1.40, *p*-value = 0.28) (Fig. 3). There was no evidence for heterogeneity (I^2^ = 0%, *p*-value = 0.42). Using the 3 studies with available data on non-DM patients, there was no evidence of EMM (*p*-value for interaction = 0.54) (Additional file 1: Figure S1).

**Fig. 4:**
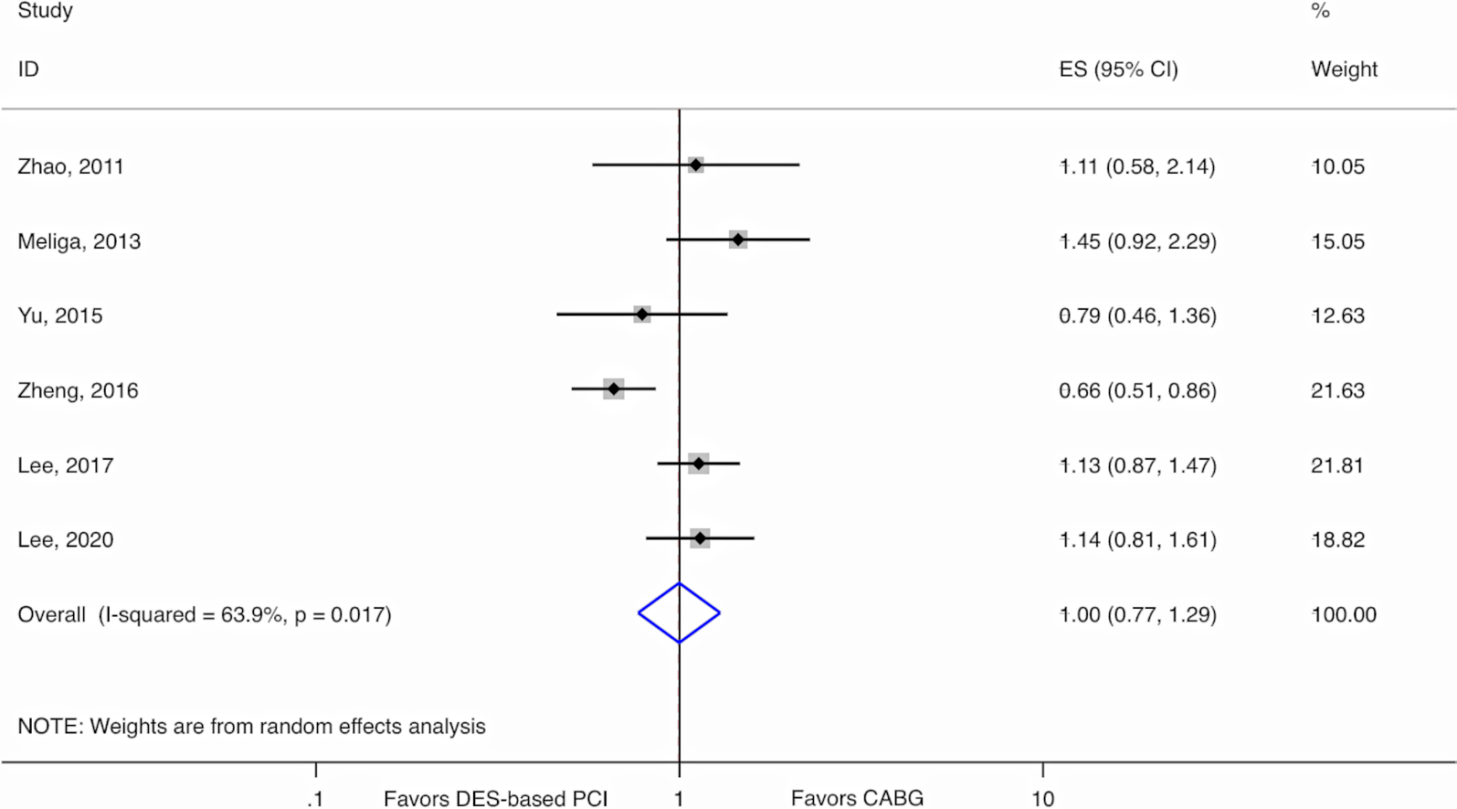
Random-effects meta-analysis for DM patients comparing DES to CABG using relative risks for the composite endpoint of all-cause mortality, myocardial infarction, or stroke in observational studies. CABG, Coronary artery bypass graft; CI, Confidence interval; DES, Drug-eluting stents; ES, Estimate

At the longest follow-up of all 6 observational studies, there was no difference between DM patients who underwent PCI with DES compared to CABG for LMCAD for the composite of all-cause mortality, myocardial infarction, or stroke (RR 1.00, 95% CI 0.77–1.29, *p*-value = 0.99) (Fig. 4). However, we observed substantial heterogeneity (I^2^ = 63.9%, *p*-value = 0.02). In the pooled analysis of the four studies with available data on non-DM patients, no evidence of EMM by diabetes was found for DES-based PCI versus CABG for this composite endpoint (*p*-value for interaction = 0.58) (Additional file 1: Figure S2).

**Fig. 5:**
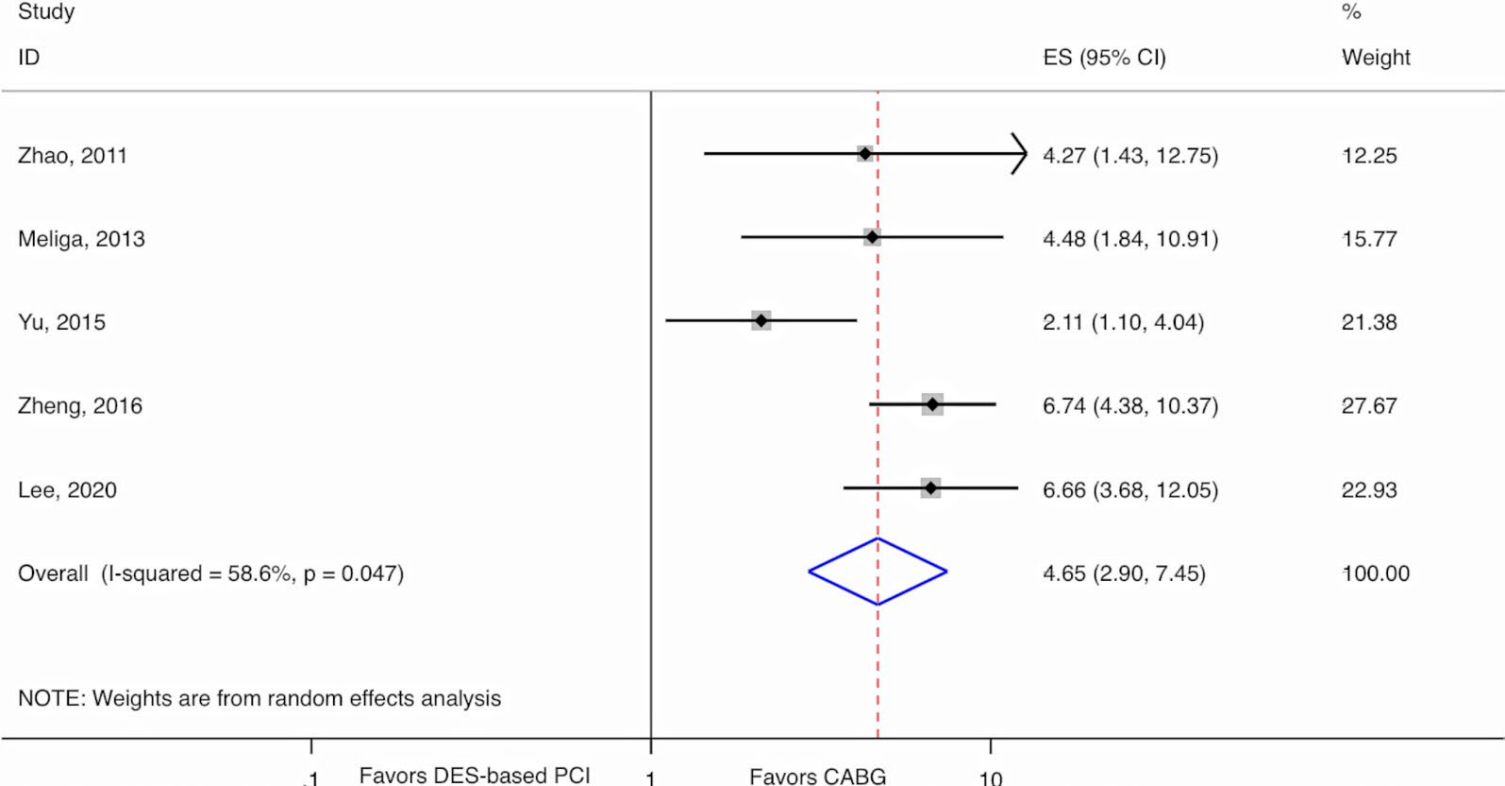
Random-effects meta-analysis for diabetic patients comparing DES to CABG using relative risks for unplanned revascularization in observational studies. CABG, Coronary artery bypass graft; CI, Confidence interval; DES, Drug-eluting stents; ES, Estimate

Using the 5 studies with available individual unplanned revascularization outcomes, the risk of unplanned revascularization among DM patients who underwent DES-based PCI was 4.5 times higher than for patients who underwent CABG (RR = 4.65, 95% CI 2.90–7.45, *p*-value =  < 0.0001) (Fig. 5). We observed moderate to substantial heterogeneity (I^2^ = 58.6%, *p*-value = 0.05). With the 3 available studies with effect estimates for non-DM patients, the wide confidence intervals of both groups overlapped (*p*-value for interaction = 0.68) (Additional file 1: Figure S3).

### Sensitivity analyses

For the RCTs, the fixed-effects meta-analysis generated nearly identical results compared to the random effects analysis (Additional file 1: Figure S4). For the observational studies, the leave-one-out analysis demonstrated that while the Zheng et al. study had the most influence, not one study significantly altered the results (Additional file 1: Figure S5). On visual inspection of the funnel plot for the composite outcome of all-cause mortality, myocardial infarction, or stroke, no evidence for significant publication bias was observed (Egger’s *p*-value = 0.60) (Additional file 1: Figure S6).

The meta-regression for follow-up time (slope = 0.01, *p*-value = 0.87), proportion of three-vessel disease for PCI patients (slope = 0.76, *p*-value = 0.56), and clinical presentation with ACS (slope = 0.58, *p*-value = 0.58) for the composite outcome of all-cause mortality, myocardial infarction, or stroke in DM patients were all non-significant in the observational studies.

## Discussion

Our study aims to shed insight on the impact of diabetes on clinical outcomes after DES-based PCI versus CABG for LMCAD. Based on RCT data, we demonstrated that DES-based PCI was inferior compared to CABG for the composite of all-cause mortality, myocardial infarction, stroke, or unplanned revascularization irrespective of DM status with a mean weighted follow-up of 4.9 years. Our data suggests that diabetes was not an effect modifier. There was no difference between DES-based PCI and CABG for all-cause mortality with a weighted follow-up of 5.2 years in the observational studies. Our meta-analysis is the first leveraging data from DM and non-DM patients from the four largest randomized controlled LMCAD revascularization trials and data from observational studies with restriction to include only DES [12]. These data may aid revascularization modality decision-making for heart teams treating DM patients with LMCAD.

Our finding that DES was inferior to CABG for DM patients for MACCE is consistent with the average population effects of all four RCTs [6–9]. Of note, the EXCEL and Nordic-Baltic-British left main revascularization study (NOBLE) trials performed randomization stratified according to the presence of diabetes, the Synergy between PCI with Taxus and Cardiac Surgery (SYNTAX) trial performed randomization stratified according to LMCAD and diabetes, while the Premier of Randomized Comparison of Bypass Surgery versus Angioplasty Using Sirolimus-Eluting Stent in Patients with Left Main Coronary Artery Disease (PRECOMBAT) trial did not stratify on diabetes status [6–9]. All four trials had pre-specified subgroup analyses comparing DM to non-DM patients that were negative for interaction. However, each trial was underpowered to detect EMM. The largest trial (EXCEL) had only 390 MACCE (248 for DM patients and 142 for non-DM patients) [8, 23]. With the caveat that EMM is a low-power analysis compared to testing for main effects, we were able to accrue nearly a threefold increase in MACCE with 1058 in total (331 in DM patients and 727 in non-DM patients) [31]. Furthermore, as the effect estimates were nearly identical for the diabetic and non-diabetic patients, this suggests, even in the presence of potentially suboptimal power, that diabetes was not a strong effect modifier of the association of revascularization strategy and outcome.

Studies have demonstrated that DM patients are more likely to have more diffuse disease, greater atherosclerotic burden, and increased lipid-rich plaques than non-DM patients [32, 33]. Furthermore, patients with DM are at higher risk of stent restenosis due to an exaggerated cellular and matrix proliferation response [34]. In the overall population, all-cause mortality after CABG seems to be lower than after PCI in patients with a high SYNTAX score of ≥ 33 [35–37]. However, there appears to be no difference in mortality in patients with low SYNTAX scores of less than 33 [37–40]. The mean weighted SYNTAX score from our analysis was in the lower range of the intermediate category. Our data suggest that diabetes may play a less critical role in patients with smaller burdens of coronary artery disease, even if the left main artery is involved.

The main reason for including observational studies in our study, apart from obtaining a total body of evidence, was to analyze the individual components of MACCE that were not available from study-level data of the RCTs. In observational studies, surgical ineligibility dictating treatment selection is likely to cause selection bias and confer a worse prognosis to DES-treated patients even in the presence of multivariable adjustment [41]. However, our results demonstrated no difference between DES and CABG for all-cause mortality. Considering that unplanned revascularization rates are the main drivers of the difference in MACCE between DES-based PCI and CABG in RCTs, these results are compatible with our analysis of RCTs [42]. Furthermore, this is consistent with the average population effects reported in the RCTs and a recent meta-analysis [5–9].

The present meta-analysis has several limitations. First, it must be highlighted that our results should be considered as hypothesis-generating, as null findings may be a function of lack of power. However, the four included RCTs demonstrated consistent results, supporting our conclusions. Second, individual studies used different definitions for the endpoints, especially for myocardial infarction and unplanned revascularization. While much information is available regarding outcome definitions for RCTs, observational studies provided less detail and may have been a source of heterogeneity for outcomes other than all-cause mortality. Third, several observational studies had violations of the proportional hazards assumption as CABG may provide more significant benefit during longer follow-ups [24, 25, 27, 29]. However, calculating the restricted mean survival time was not possible without the published adjusted survival curves or individual participant data [43]. Finally, the PCI technique for LMCAD is a rapidly evolving field, and categorizing PCI as DES-based or BMS-based could be seen as simplistic. The impact of intravascular imaging, newer DES platforms with increased radial strength, and different bifurcation strategies could have a clinical effect beyond whether DES was used when PCI is compared to CABG.

## Conclusion

In RCTs, DES-based PCI was associated with an increased risk in the composite endpoint of all-cause mortality, myocardial infarction, stroke, and unplanned revascularization compared to CABG in LMCAD patients with or without diabetes. No EMM by DM status was observed. Furthermore, there was no difference for all-cause mortality in the observational studies. Heart teams may consider DM as one of the many components in the clinical decision-making process, but may not want to consider DM as a primary deciding factor between DES-based PCI and CABG for LMCAD with low to intermediate anatomical complexity in the other coronary arteries.

## Supplementary Information

**Additional file 1. Table S1**. The PRISMA 2020 27-item checklist for reporting in systematic reviews and meta-analyses. **Table S2**. The detailed search strategy for PubMed, Embase and the Cochrane Central Register of Controlled Trials (CENTRAL).** Table S3**. Detailed definitions of key outcomes from the four included randomized controlled trials (Morice et al. 2014, Milojevic et al. 2019, Holm et al. 2020, and Park et al. 2020).** Table S4**. Detailed evaluation of the risk of bias for the randomized controlled trials using the Cochrane’s Collaboration risk-of-bias (RoB 2) tool.** Table S5**. Detailed evaluation of the risk of bias for the observational studies using the Risk of Bias in Non-randomized Studies of Interventions (ROBINS-I) tool.** Figure S1**. Random-effects meta-analysis testing for effect measure modification by diabetes comparing DES to CABG using relative risks for all-cause mortality. 1=DM, 0 = non-DM; ES, estimate; CI, confidence interval.** Figure S2**. Random-effects meta-analysis testing for effect measure modification by diabetes comparing DES to CABG using relative risks for all-cause mortality, myocardial infarction, or stroke. 1 = DM, 0 = non-DM; ES, estimate; CI, confidence interval.** Figure S3**. Random-effects meta-analysis testing for effect measure modification by diabetes comparing DES to CABG using relative risks for revascularization. 1 = DM, 0 = non-DM; ES, estimate; CI, confidence interval.** Figure S4**. Fixed effects meta-analysis comparing DES to CABG in diabetic patients using relative risks for the composite endpoint of all-cause mortality, myocardial infarction, stroke, or unplanned revascularization. 1 = DM, 0 = non-DM; ES, estimate; CI, confidence interval.** Figure S5**. Influence analysis with each study being excluded in turn. 1, Zhao 2011; 2, Meliga 2013; 3, Yu 2015; 4, Zheng 2016; 5, Lee 2017; 6, Lee 2020. Figure S6: Funnel plot of the observational studies for the composite endpoint of all-cause mortality, myocardial infarction, or stroke.

## Data Availability

The data that support the study findings are available from the online publication databases as mentioned in the manuscript.

## Abbreviations

ACS: Acute coronary syndrome
BMS: Bare-metal stents
CABG: Coronary artery bypass graft
CI: Confidence intervals
DES: Drug-eluting stents
DM: Diabetes mellitus
EMM: Effect-measure modification
LMCAD: Left main coronary artery disease
MACCE: Major adverse cardiovascular and cerebrovascular events
PCI: Percutaneous coronary intervention
RCT: Randomized controlled trial
RR: Relative risk
