# Supplementary material for: The effect of diabetes on surgical versus percutaneous left main revascularization outcomes: a systematic review and meta-analysis
Source: J Cardiothorac Surg. 2022 Apr 1;17:61. doi: 10.1186/s13019-022-01795-w (PMC8973812; doi:10.1186/s13019-022-01795-w)
Supplement: Supplementary file 1 — Additional file 1. Table S1. The PRISMA 2020 27-item checklist for reporting in systematic reviews and meta-analyses. Table S2. The detailed search strategy for PubMed, Embase and the Cochrane Central Register of Controlled Trials (CENTRAL). Table S3. Detailed definitions of key outcomes from the four included randomized controlled trials (Morice et al. 2014, Milojevic et al. 2019, Holm et al. 2020, and Park et al. 2020). Table S4. Detailed evaluation of the risk of bias for the randomized controlled trials using the Cochrane’s Collaboration risk-of-bias (RoB 2) tool. Table S5. Detailed evaluation of the risk of bias for the observational studies using the Risk of Bias in Non-randomized Studies of Interventions (ROBINS-I) tool. Figure S1. Random-effects meta-analysis testing for effect measure modification by diabetes comparing DES to CABG using relative risks for all-cause mortality. 1=DM, 0 = non-DM; ES, estimate; CI, confidence interval. Figure S2. Random-effects meta-analysis testing for effect measure modification by diabetes comparing DES to CABG using relative risks for all-cause mortality, myocardial infarction, or stroke. 1 = DM, 0 = non-DM; ES, estimate; CI, confidence interval. Figure S3. Random-effects meta-analysis testing for effect measure modification by diabetes comparing DES to CABG using relative risks for revascularization. 1 = DM, 0 = non-DM; ES, estimate; CI, confidence interval. Figure S4. Fixed effects meta-analysis comparing DES to CABG in diabetic patients using relative risks for the composite endpoint of all-cause mortality, myocardial infarction, stroke, or unplanned revascularization. 1 = DM, 0 = non-DM; ES, estimate; CI, confidence interval. Figure S5. Influence analysis with each study being excluded in turn. 1, Zhao 2011; 2, Meliga 2013; 3, Yu 2015; 4, Zheng 2016; 5, Lee 2017; 6, Lee 2020. Figure S6: Funnel plot of the observational studies for the composite endpoint of all-cause mortality, myocardial infarction, or stroke. [file 13019_2022_1795_MOESM1_ESM.docx]

**The Effect of Diabetes on Surgical versus Percutaneous Left Main Revascularization Outcomes: A Systematic Review and Meta-Analysis**

**Supplementary Materials**

**Table S1. PRISMA 2020 Checklist**

| **Section and Topic** | **Item #** | **Checklist item** | **Location where item is reported** |
| --- | --- | --- | --- |
| **TITLE** | | |  |
| Title | 1 | Identify the report as a systematic review. | p. 1 |
| **ABSTRACT** | | |  |
| Abstract | 2 | See the PRISMA 2020 for Abstracts checklist. | p. 3 |
| **INTRODUCTION** | | |  |
| Rationale | 3 | Describe the rationale for the review in the context of existing knowledge. | p. 4 |
| Objectives | 4 | Provide an explicit statement of the objective(s) or question(s) the review addresses. | p. 4 |
| **METHODS** | | |  |
| Eligibility criteria | 5 | Specify the inclusion and exclusion criteria for the review and how studies were grouped for the syntheses. | p. 5 |
| Information sources | 6 | Specify all databases, registers, websites, organisations, reference lists and other sources searched or consulted to identify studies. Specify the date when each source was last searched or consulted. | p. 5 |
| Search strategy | 7 | Present the full search strategies for all databases, registers and websites, including any filters and limits used. | Table S2 |
| Selection process | 8 | Specify the methods used to decide whether a study met the inclusion criteria of the review, including how many reviewers screened each record and each report retrieved, whether they worked independently, and if applicable, details of automation tools used in the process. | p. 5-6 |
| Data collection process | 9 | Specify the methods used to collect data from reports, including how many reviewers collected data from each report, whether they worked independently, any processes for obtaining or confirming data from study investigators, and if applicable, details of automation tools used in the process. | p. 5-6 |
| Data items | 10a | List and define all outcomes for which data were sought. Specify whether all results that were compatible with each outcome domain in each study were sought (e.g. for all measures, time points, analyses), and if not, the methods used to decide which results to collect. | p. 5-6 |
|  | 10b | List and define all other variables for which data were sought (e.g. participant and intervention characteristics, funding sources). Describe any assumptions made about any missing or unclear information. | p. 5-6 |
| Study risk of bias assessment | 11 | Specify the methods used to assess risk of bias in the included studies, including details of the tool(s) used, how many reviewers assessed each study and whether they worked independently, and if applicable, details of automation tools used in the process. | p. 5-6 |
| Effect measures | 12 | Specify for each outcome the effect measure(s) (e.g. risk ratio, mean difference) used in the synthesis or presentation of results. | p. 7 |
| Synthesis methods | 13a | Describe the processes used to decide which studies were eligible for each synthesis (e.g. tabulating the study intervention characteristics and comparing against the planned groups for each synthesis (item #5)). | p. 7 |
|  | 13b | Describe any methods required to prepare the data for presentation or synthesis, such as handling of missing summary statistics, or data conversions. | p. 7 |
|  | 13c | Describe any methods used to tabulate or visually display results of individual studies and syntheses. | p. 7 |
|  | 13d | Describe any methods used to synthesize results and provide a rationale for the choice(s). If meta-analysis was performed, describe the model(s), method(s) to identify the presence and extent of statistical heterogeneity, and software package(s) used. | p. 7 |
|  | 13e | Describe any methods used to explore possible causes of heterogeneity among study results (e.g. subgroup analysis, meta-regression). | p. 7 |
|  | 13f | Describe any sensitivity analyses conducted to assess robustness of the synthesized results. | p. 7 |
| Reporting bias assessment | 14 | Describe any methods used to assess risk of bias due to missing results in a synthesis (arising from reporting biases). | p. 7 |
| Certainty assessment | 15 | Describe any methods used to assess certainty (or confidence) in the body of evidence for an outcome. | p. 7 |
| **RESULTS** | | |  |
| Study selection | 16a | Describe the results of the search and selection process, from the number of records identified in the search to the number of studies included in the review, ideally using a flow diagram. | Figure 1 |
|  | 16b | Cite studies that might appear to meet the inclusion criteria, but which were excluded, and explain why they were excluded. | p. 9 |
| Study characteristics | 17 | Cite each included study and present its characteristics. | Table 1 |
| Risk of bias in studies | 18 | Present assessments of risk of bias for each included study. | Tables S4 and S5 |
| Results of individual studies | 19 | For all outcomes, present, for each study: (a) summary statistics for each group (where appropriate) and (b) an effect estimate and its precision (e.g. confidence/credible interval), ideally using structured tables or plots. | Figures 2-5 and Figures S1-S5 |
| Results of syntheses | 20a | For each synthesis, briefly summarise the characteristics and risk of bias among contributing studies. | p. 8-9 |
|  | 20b | Present results of all statistical syntheses conducted. If meta-analysis was done, present for each the summary estimate and its precision (e.g. confidence/credible interval) and measures of statistical heterogeneity. If comparing groups, describe the direction of the effect. | p. 8-10 |
|  | 20c | Present results of all investigations of possible causes of heterogeneity among study results. | p. 8-10 |
|  | 20d | Present results of all sensitivity analyses conducted to assess the robustness of the synthesized results. | p. 8-10 |
| Reporting biases | 21 | Present assessments of risk of bias due to missing results (arising from reporting biases) for each synthesis assessed. | Figure S6 |
| Certainty of evidence | 22 | Present assessments of certainty (or confidence) in the body of evidence for each outcome assessed. | Tables S4-S5 |
| **DISCUSSION** | | |  |
| Discussion | 23a | Provide a general interpretation of the results in the context of other evidence. | p. 11-14 |
|  | 23b | Discuss any limitations of the evidence included in the review. | p. 11-14 |
|  | 23c | Discuss any limitations of the review processes used. | p. 11-14 |
|  | 23d | Discuss implications of the results for practice, policy, and future research. | p. 11-14 |
| **OTHER INFORMATION** | | |  |
| Registration and protocol | 24a | Provide registration information for the review, including register name and registration number, or state that the review was not registered. | p. 5 |
|  | 24b | Indicate where the review protocol can be accessed, or state that a protocol was not prepared. | p. 5 |
|  | 24c | Describe and explain any amendments to information provided at registration or in the protocol. | p. 5-6 |
| Support | 25 | Describe sources of financial or non-financial support for the review, and the role of the funders or sponsors in the review. | p. 5-6 |
| Competing interests | 26 | Declare any competing interests of review authors. | p. 14 |
| Availability of data, code and other materials | 27 | Report which of the following are publicly available and where they can be found: template data collection forms; data extracted from included studies; data used for all analyses; analytic code; any other materials used in the review. | p.5-7 |

*From:*  Page MJ, McKenzie JE, Bossuyt PM, Boutron I, Hoffmann TC, Mulrow CD, et al. The PRISMA 2020 statement: an updated guideline for reporting systematic reviews. BMJ 2021;372:n71. doi: 10.1136/bmj.n71

For more information, visit: <http://www.prisma-statement.org/>

**Table S2. Search strategy**

| **Pubmed** | |
| --- | --- |
| Search strategy | ("Percutaneous Coronary Intervention"[Mesh] OR percutaneous coronary intervention*[tiab] OR pci[tiab] OR "Drug-Eluting Stents"[Mesh] OR eluting*[tiab] OR des[tiab])  AND  ("Coronary Artery Bypass"[Mesh] OR bypass*[tiab] OR CABG[tiab])  AND  (left main[tiab] OR lmca[tiab] OR lmt[tiab]) |
| Restrictions | Language: English language only  Dates: January 1, 1999 to March 1, 2021 |
| Results | With the search strategy and restriction, we obtained 1,486 results on March 1, 2021. |
| **EMBASE** | |
| Search strategy | ('percutaneous coronary intervention'/exp OR 'drug eluting stent'/exp OR 'percutaneous coronary intervention*':ab,ti,kw OR pci:ab,ti,kw OR eluting*:ab,ti,kw OR des:ab,ti,kw) AND [embase]/lim  AND  ('coronary artery bypass graft'/exp OR bypass*:ab,ti,kw OR CABG:ab,ti,kw) AND [embase]/lim  AND  ('left coronary artery'/exp OR left main:ab,ti,kw OR lmca:ab,ti,kw OR lmt:ab,ti,kw) AND [embase]/lim |
| Restrictions | Language: English language only  Dates: January 1, 1999, to March 1, 2021 |
| Results | With the search strategy and restriction, we obtained 1,774 results on March 1, 2021. |
| **Cochrane Central Register of Controlled Trials** | |
| Search strategy | "Drug-Eluting Stents"[Mesh] AND "Coronary Artery Bypass"[Mesh] |
| Restrictions | Language: English language only  Dates: January 1, 1999, to March 1, 2021 |
| Results | With the search strategy and restrictions, we obtained 107 results on March 1, 2021. |

**Table S3. Definitions of RCT outcomes**

| **Study** | **Outcome definition** |
| --- | --- |
| Morice, 2014 | **All-cause mortality**  All death, including cardiovascular death, non-cardiovascular death, and death of undetermined cause.  **Myocardial infarction (MI)**  Spontaneous MI: Defined as a new electrocardiogram (EKG) Q wave, or enzyme change of more than 10% of CK-MB/total-CK ratio or CK-MB of at least 5 times the upper limit of normal (ULN) at least 7 days after the index revascularization.  Procedural MI: Defined as above, but within 7 days of the index revascularization.  **Stroke**  Acute neurological event leading to irreversible brain damage related to the impairment of cerebral circulation lasting more than 24 hours as evaluated by a neurologist.  **Repeat revascularization**  Any revascularization by PCI or CABG. |
| Milojevic, 2019 | **All-cause mortality**  All death, including cardiovascular death, non-cardiovascular death, and death of undetermined cause.  **Myocardial infarction**  Spontaneous MI: Defined as troponin or CK-MB value > 1x ULN with EKG ST changes, Q waves, angiographic changes, or imaging changes >72 hours after the index revascularization.  Procedural MI: Defined as CK-MB >10xULN, or >5xULN with Q waves, angiographic changes, or imaging changes within 72 hours of index revascularization.  **Stroke**  Stroke was defined as a rapid-onset, new, persistent neurologic deficit attributed to cerebral blood flow obstruction or hemorrhage (as determined by a vascular neurologist or stroke specialist). The stroke outcome had 4 criteria:   1. New-onset neurological symptoms and signs suggestive of stroke. 2. Duration of more than 24 hours or less than 24 hours with intervention with suggestive imaging changes or death due to neurological deficit. 3. No alternative explanation. 4. Neurologist diagnosis using imaging or lumbar puncture.   **Ischemia-driven revascularization**  PCI or CABG to lesion with more than 50% diameter stenosis by quantitative coronary angiography and one of the following:   1. Positive physiological study suggestive of ischemia in the target lesion territory. 2. EKG changes suggestive of ischemia in the target lesion territory. 3. Typical ischemic symptoms. 4. Positive intracoronary imaging (intravascular ultrasound) or positive intracoronary physiological testing (fractional flow reserve). |
| Holm, 2020 | **All-cause mortality**  All death, including cardiovascular death, non-cardiovascular death, and death of undetermined cause.  **Myocardial infarction**  Spontaneous MI: Defined as troponin values of more than >1x the ULN with either significant angiographic changes, ischemic symptoms, imaging changes, or ST/Q wave changes. Of note, procedural MI was not included in the primary outcome.  **Stroke**  Ischemic or hemorrhagic stroke verified by CT or MRI.  **Repeat revascularization**  Any CABG or PCI during follow-up. |
| Park, 2020 | **All-cause mortality**  All death, including cardiovascular death, non-cardiovascular death, and death of undetermined cause.  **Myocardial infarction**  Spontaneous MI: Defined as typical troponin changes (or more rapid for CK-MB) >48 hours after index revascularization with at least one of the following:   1. Enzyme changes (CK-MB >1x ULN). 2. Ischemic symptoms. 3. New Q waves.   Procedural MI: Defined as new Q waves and CK-MB at least 5x ULN within 48h of index revascularization.  **Stroke**  Sudden onset symptoms consistent with stroke lasting >24 hours.  **Ischemia-driven revascularization**  PCI or CABG to index lesion with ≥50% diameter stenosis by quantitative coronary angiography and any of the following:   1. Positive physiological study in the in-index lesion territory. 2. EKG changes at rest in the index lesion territory. 3. Ischemic symptoms. |

**Table S4. Risk of bias assessment for RCTs using the Cochrane’s Collaboration risk-of-bias tool (RoB 2)**

| **Study** | **Randomization process** | **Deviations from the intended interventions** | **Missing outcome data** | **Measurement of outcome** | **Selection of reported result** | **Overall bias** |
| --- | --- | --- | --- | --- | --- | --- |
| **Morice, 2014** | **Low risk**  Randomization was stratified for LMCAD and diabetes. | **Low risk**  Unblinded, however, minimal deviations from intended interventions. | **Low risk**  From the time of randomization to 5-year follow-up, a total of 92.5% and 96.9% of patients were available in the CABG and DES groups, respectively. | **Some concerns**  The outcomes were adjudicated by an independent clinical events committee (cardiologists, surgeons, and neurologists), however, blinding was not specified except for stroke. | **Low risk**  Subgroup analyses were pre-specified. | **Some concern**  The absence of blinded adjudication of clinical events may diminish the quality of this study regarding the validity of bias-susceptible outcomes. |
| **Milojevic, 2019** | **Low risk**  Randomization was stratified for diabetes. | **Low risk**  Unblinded, however, minimal deviations from intended interventions. | **Low risk**  More than 90% follow-up at the 5-year study with only 3.1% difference between groups. | **Some concerns**  The outcomes were adjudicated by an independent clinical events committee (cardiologists, surgeons, and neurologists), however, blinding was not specified except for stroke | **Low risk**  This analysis was a prespecified sub-study. | **Some concern**  The absence of blinded adjudication of clinical events may diminish the quality of this study regarding the validity of bias-susceptible outcomes. |
| **Holm, 2020** | **Low risk**  Randomization was stratified for diabetes. | **Some concerns**  Unblinded, the surgeon or cardiologist could overrule the assignment if the patient was found not to be eligible or if the patient refused. However, only 13 patients did not receive PCI, and only 33 patients did not receive CABG after randomization. | **Low risk**  Only 6 patients and 11 patients in the PCI and CABG groups were lost to follow-up, which results in > 98% follow-up. | **Come concerns**  Independent clinical events committee, blinding unspecified. | **Low risk**  Subgroup analyses were pre-specified. | **Some concern**  The absence of blinded adjudication and possible bias allocation raises some concerns for this study. |
| **Park, 2020** | **Some concern**  Randomization was not stratified for diabetes. | **Low risk**  Unblinded, however, minimal deviations from intended interventions. | **Low risk**  Ten-year follow-up for all clinical endpoints was achieved in 96% of patients randomized to PCI and CABG. | **Low risk**  Independent blinded adjudication committee. | **Low risk**  Subgroup analyses were pre-specified. | **Some concern**  The absence of stratification for LMCAD and diabetes during the randomization process resulted in less robust subgroup analyses. |

**Table S5. Risk of bias assessment for observational studies using the ROBINS-I tool**

| **Study** | **Confounding** | **Selection of participants** | **Classification of intervention** | **Deviations from intended interventions** | **Missing data** | **Measurements of outcomes** | **Reported results** | **Overall risk of bias** |
| --- | --- | --- | --- | --- | --- | --- | --- | --- |
| **Zhao, 2011** | **Serious**  Multivariable adjustment does not account for a significant number of potential confounders. | **Serious**  Risk of selection bias favoring CABG. However, outcomes of interest were not present at the start of the study. Consecutive patient selection. Unclear if the study population is comprised of consecutive patients. | **Low**  Interventions clearly defined. | **Low**  The interventions are one-time procedures. | **Low**  Long-term outcomes were available in 98.2% for the PCI group and 96.6% for the CABG group. | **Moderate**  Unblinded physician adjudication of events. MI, stroke, and revascularization may be prone to ascertainment bias. | **Moderate**  Prespecified statistical analysis plan not available. | **Serious**  Suboptimal confounder adjustment and potential selection bias are the main limitations regarding the validity of the effect estimates. |
| **Meliga, 2013** | **Moderate**  Reasonable confounding adjustment using propensity scores. | **Moderate**  Risk of selection bias favoring CABG. However, outcomes of interest were not present at the start of the study. Consecutive patient selection. | **Low**  Interventions clearly defined. | **Low**  The interventions are one-time procedures. | **Low**  Long-term outcomes in 98.9% of the total study population. | **Moderate**  Neurologist adjudicated stroke. Other outcomes were well-defined, but the adjudication process is unclear. | **Moderate**  Prespecified statistical analysis plan not available. | **Moderate**  Potential selection bias may affect the validity of the estimates. |
| **Yu, 2015** | **Serious**  Multivariable adjustment does not account for a significant number of potential confounders. | **Moderate**  Risk of selection bias favoring CABG. However, outcomes of interest were not present at the start of the study. Consecutive patient selection. | **Low**  Interventions clearly defined. | **Low**  The interventions are one-time procedures. | **Low**  Complete follow-up was available in 93.2% of the total study population. | **Moderate**  Unblinded physician adjudication of events. MI, stroke, and revascularization may be prone to ascertainment bias. | **Moderate**  Prespecified statistical analysis plan not available. | **Serious**  Suboptimal confounder adjustment and potential selection bias are the main limitations regarding the validity of the effect estimates. |
| **Zheng, 2016** | **Moderate**  Reasonable confounding adjustment using propensity scores. | **Moderate**  Risk of selection bias favoring CABG. However, outcomes of interest were not present at the start of the study. Consecutive patient selection. | **Low**  Interventions clearly defined. | **Low**  The interventions are one-time procedures. | **Low**  Complete follow-up in 98.9% of the overall study population. | **Moderate**  Unclear if blinded. Adjudicated by an independent committee of physicians. MI, stroke, and revascularization may be prone to ascertainment bias. | **Moderate**  Unclear if subgroup diabetic subgroup analyses were prespecified. | **Moderate**  Potential selection bias may affect the validity of the estimates. |
| **Lee, 2017** | **Low**  Robust adjustment by propensity score matching. | **Moderate**  Risk of selection bias favoring CABG. However, outcomes of interest were not present at the start of the study. Consecutive patient selection. | **Low**  Interventions clearly defined. | **Low**  The interventions are one-time procedures. | **No information**  Probable minimal loss to follow-up, but not explicitly stated in the study. | **Moderate**  Unclear if blinded. Adjudicated by an independent committee of physicians. MI, stroke, and revascularization may be prone to ascertainment bias. | **Moderate**  Unclear if subgroup diabetic subgroup analyses were prespecified. | **Moderate**  Potential selection bias may affect the validity of the estimates. |
| **Lee, 2020** | **Moderate**  Reasonable adjustment by inverse probability of treatment weighting. | **Moderate**  Risk of selection bias favoring CABG. However, outcomes of interest were not present at start of the study. Consecutive patient selection. | **Low**  Interventions clearly defined. | **Low**  The interventions are one-time procedures. | **No information**  Probable minimal loss to follow-up, but not explicitly stated in the study. | **Moderate**  Unclear if blinded. Adjudicated by an independent committee of physicians. MI, stroke, and revascularization may be prone to ascertainment bias. | **Moderate**  Prespecified statistical analysis plan not available. | **Moderate**  Potential selection bias may affect the validity of the estimates. |

**Figure S1.** Random-effects meta-analysis testing for effect measure modification by diabetes comparing DES to CABG using relative risks for all-cause mortality. 1=DM, 0 = non-DM; ES, estimate; CI, confidence interval

**
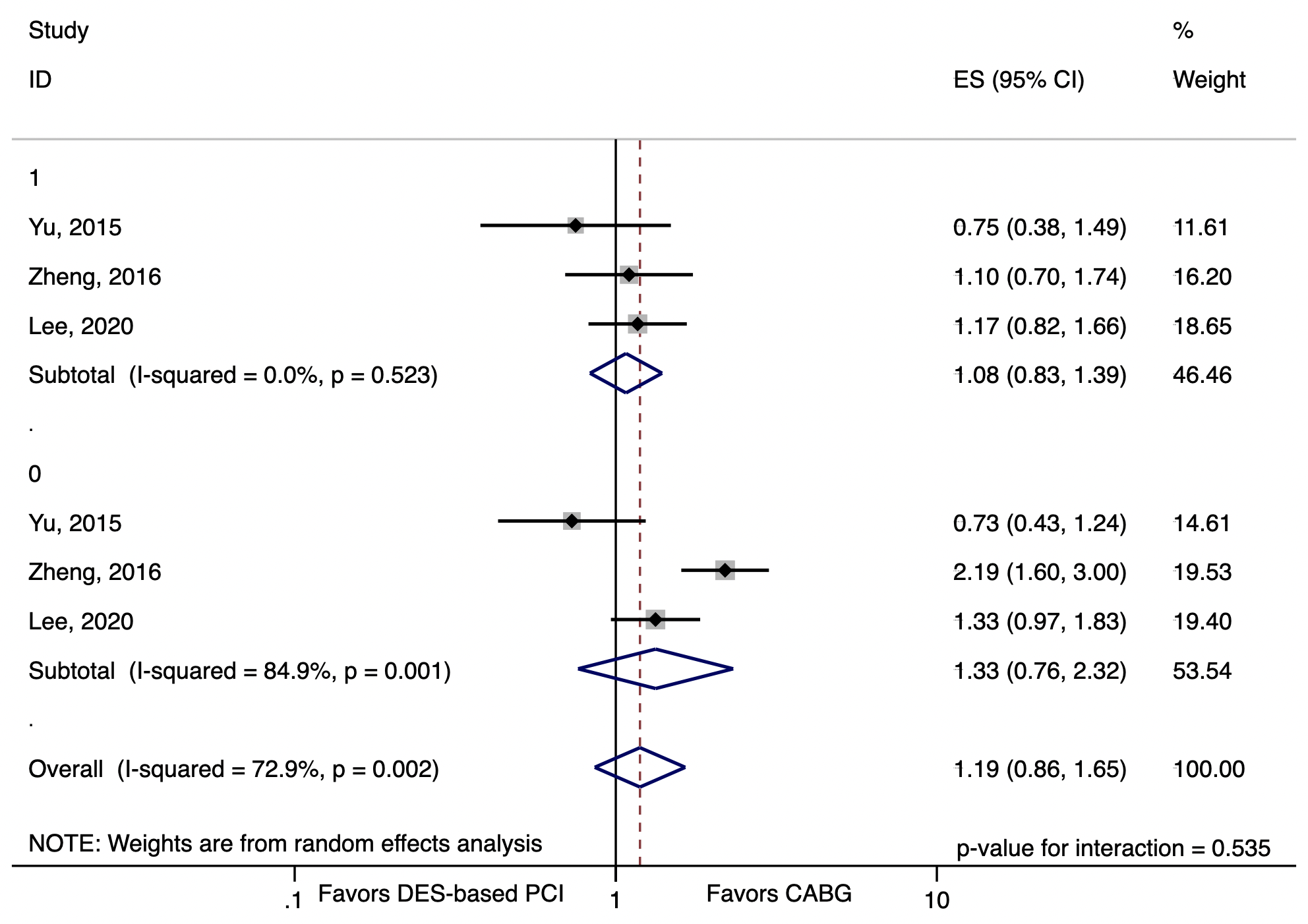
**

**Figure S2.** Random-effects meta-analysis testing for effect measure modification by diabetes comparing DES to CABG using relative risks for all-cause mortality, myocardial infarction, or stroke. 1 = DM, 0 = non-DM; ES, estimate; CI, confidence interval


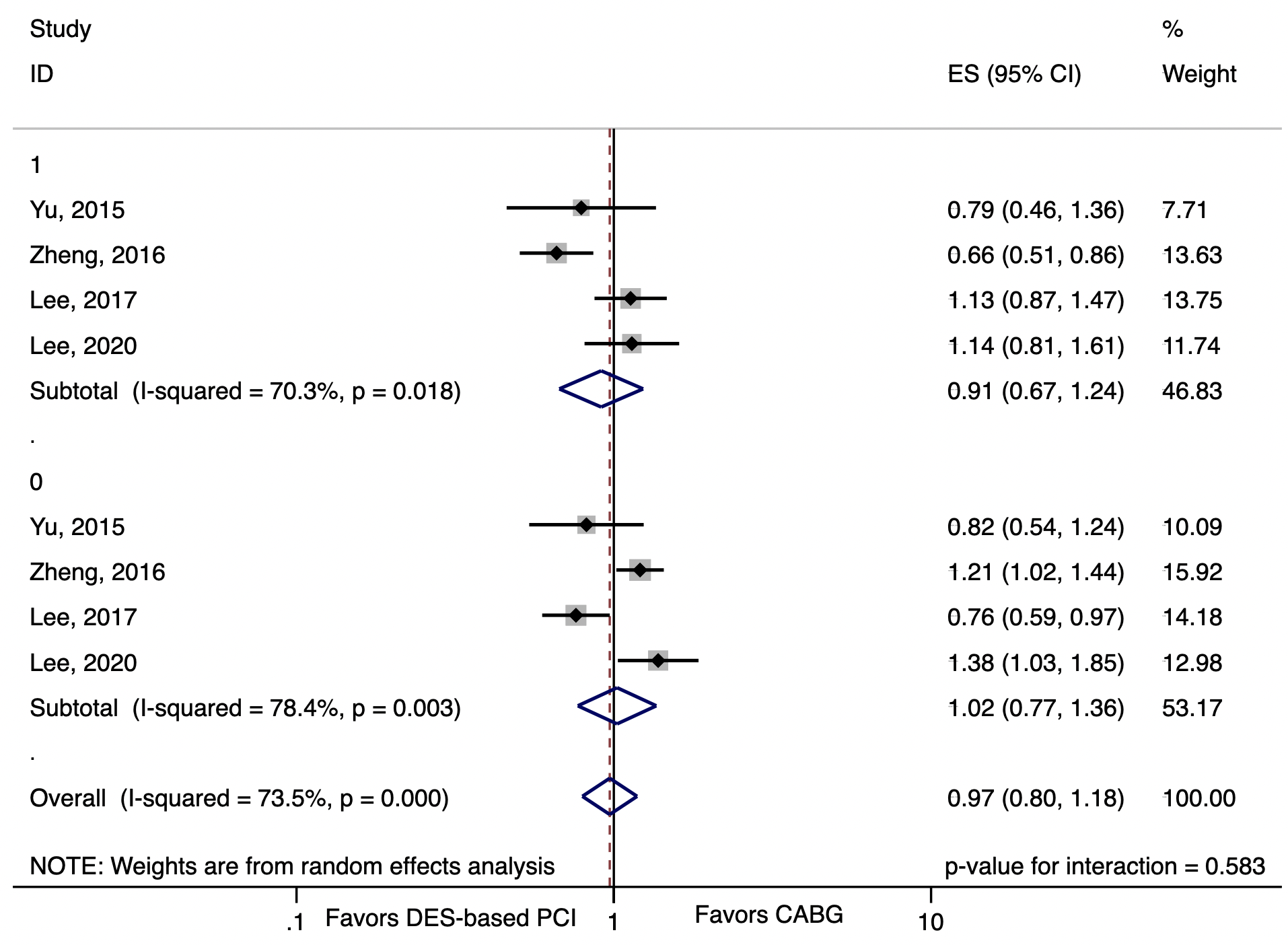


**Figure S3.** Random-effects meta-analysis testing for effect measure modification by diabetes comparing DES to CABG using relative risks for revascularization. 1 = DM, 0 = non-DM; ES, estimate; CI, confidence interval

**
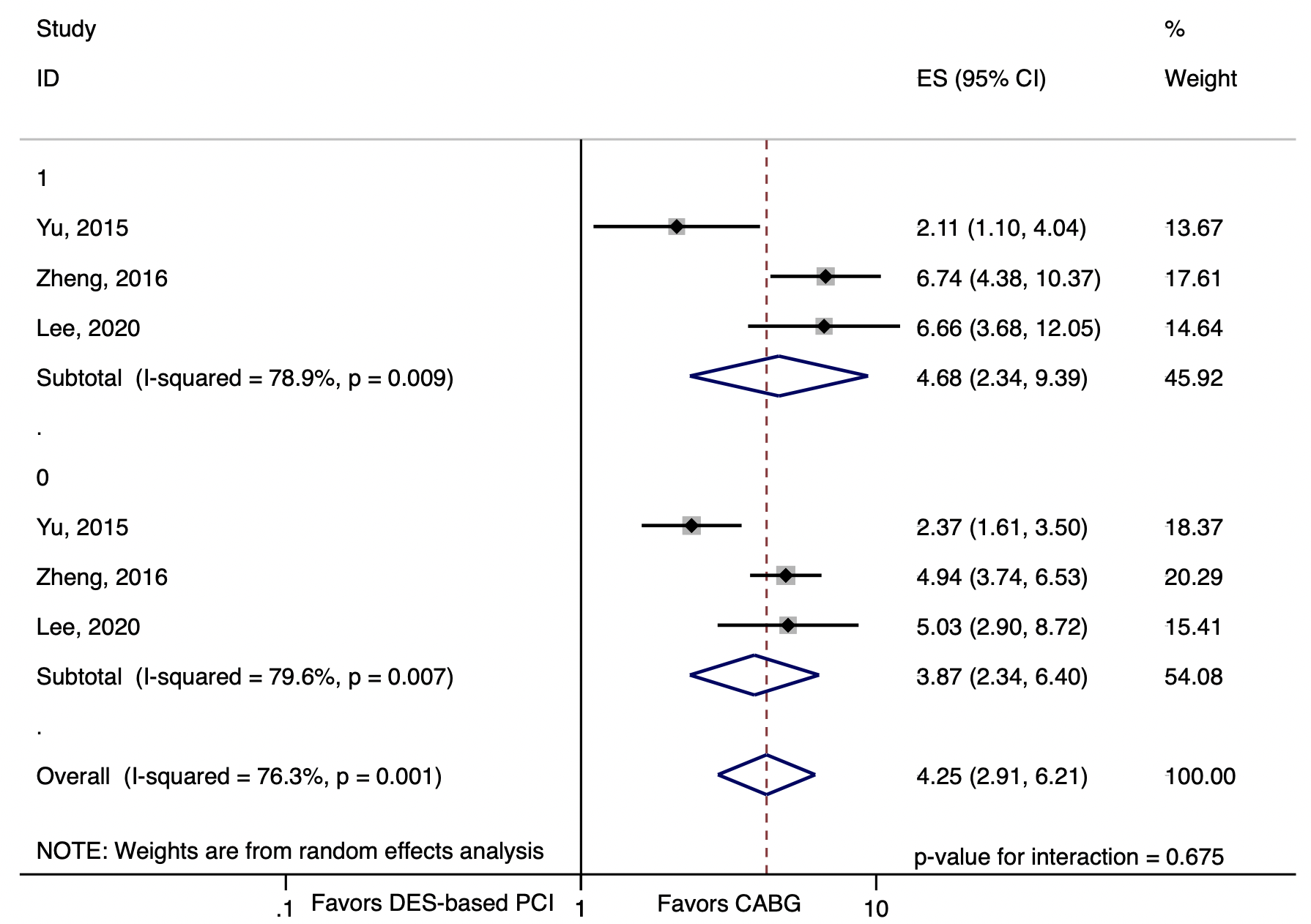
**

**Figure S4.** Fixed effects meta-analysis comparing DES to CABG in diabetic patients using relative risks for the composite endpoint of all-cause mortality, myocardial infarction, stroke, or unplanned revascularization. 1 = DM, 0 = non-DM; ES, estimate; CI, confidence interval


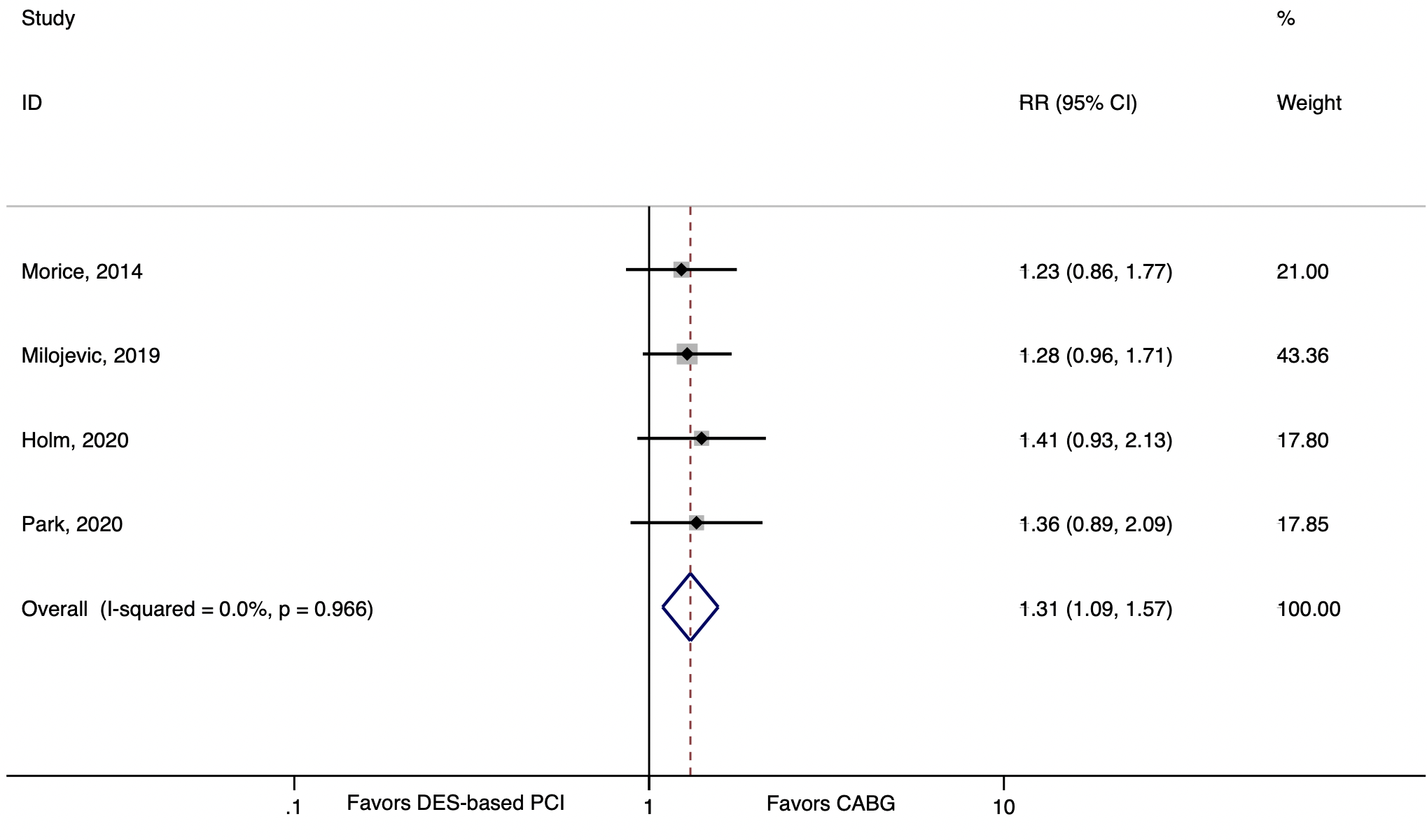


**Figure S5.** Influence analysis with each study being excluded in turn. 1, Zhao 2011; 2, Meliga 2013; 3, Yu 2015; 4, Zheng 2016; 5, Lee 2017; 6, Lee 2020.


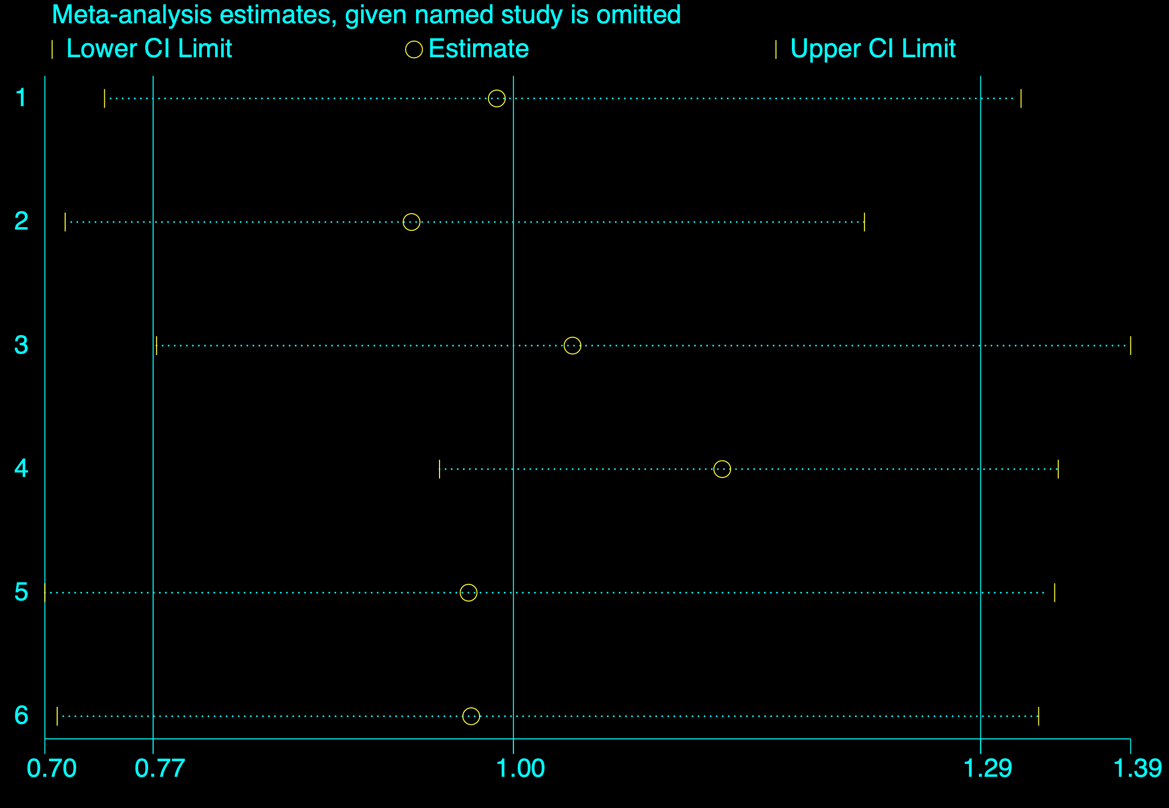


**Figure S6.** Funnel plot of the observational studies for the composite endpoint of all-cause mortality, myocardial infarction, or stroke.

**
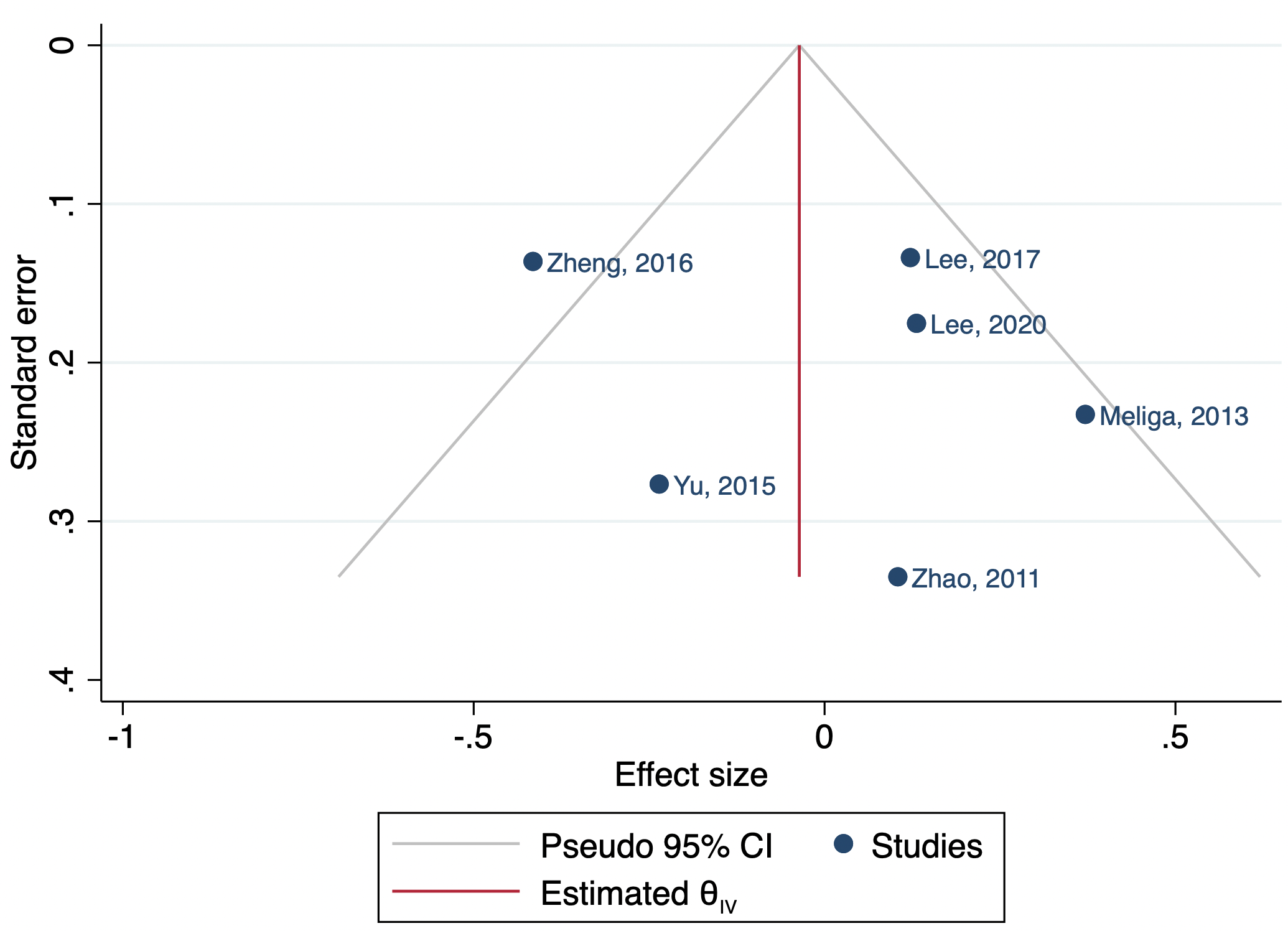
**
